# Can Syphilis Be Cured?1Read at the thirty-first annual meeting of the Medical Association of Central New York, at Auburn, October 18, 1898.

**Published:** 1899-04

**Authors:** A. E. Diehl

**Affiliations:** Buffalo, N. Y.


### CAN SYPHILIS BE CURED?[1]

BY A. E. DIEHL, M. D., Buffalo, N. Y.

CAN syphilis be cured? This is a question which has been asked since syphilis was known as such. To my mind it is a question of great importance, because syphilis is a disease whose devastations extend through all countries and all people, and while in itself it is rarely dangerous to life, yet it can produce more mental suffering and anguish than any other known disease, except probably leprosy. How many young people have contracted the disease, and later when marriage is considered and the thought comes up of the possibility of communicating the disease to one's offspring, here is where oftentimes the sufferer wishes that death might have come before. Yet much of this mental suffering can be avoided by the knowledge that syphilis can be cured. There is today a large majority of physicians who think and say that the disease is incurable and in this they are nearly right, for when it reaches a certain stage, it is incurable.

By curability of the disease we do not mean merely the disappearance of any visible evidences, but the entire elimination of it, con-

---

1. Read at the thirty-first annual meeting of the Medical Association of Central New York, at Auburn, October 18, 1898.

sequent upon the destruction of the virus or toxin, whatever it may be. In some cases it is with regret that I say the physician is at fault, for after the disappearance of all the symptoms the patient is discharged and told he is cured, but in the majority of cases it is, of course, the fault of the patient, for at the disappearance of the visible symptoms he imagines himself cured and discontinues all treatment; sometimes such cases are cured, even by a few months' treatment, but more often it happens that in after years he goes to some other physician suffering either from pronounced tertiary syphilis or some obscure visceral lesion.

The question arises, how do we know or how can we tell that the disease is cured? This unfortunately is a question which cannot be answered definitely. Again it may be asked, how do we know that syphilis is curable? This is easily answered. By a reinfection.

That syphilis is curable is demonstrated to many of us by the fact that every day we see people who had been infected in former years and today they are perfectly healthy, with absolutely no traces of the disease, and they are parents of healthy, robust children. Many cases could be cited. I will merely refer to one as an example:

A man, W., aged about 40 years, was infected 14 years ago. Shortly after, he married and communicated it to his wife. Both underwent a thorough course of treatment, and are today the parents of two healthy, robust children, one 10 years of age and the other 6 years. Neither of the parents ever showed any later symptoms of the disease.

In the earlier years of modern therapeutics such results were seen but rarely, because the disease was not so thoroughly studied and treated, but of late years both the physician and the patient have learned that results could be obtained only by long, continued and faithful treatment. How often do we see manifestations of tertiary syphilis among the present generation? I can say without fear of contradiction that gummata are becoming an uncommon lesion.

Again, it may be asked what period of time must lapse before tertiary symptoms appear? In the vast majority of cases where tertiary symptoms have appeared, they were from three to five years after infection, though, of course, they have appeared as late as twenty years and more after infection. Nevins Hyde has recently issued a publication in which he states, that between 80 per cent. and 90 per cent. of all cases of acquired syphilis never reach the tertiary stage; and as time advances and the laity become better educated to the fact that the disease can be cured by faithful and persistent treatment, this percentage will become higher. I do not wish to be understood as saying that all cases of syphilis are curable, for undoubtedly there are cases which cannot be cured, even when treatment has been begun at the earliest symptoms. Such cases and those called malignant syphilis will resist every method of treatment. The objective symptoms may be made to disappear for a short time, but will break out again and again despite our best efforts.

What do we understand the term reinfection to signify? The term is allowed only when after the course of a typical first general infection, several years later an initial lesion develops, which pursues the same course as the first one, followed by a typical universal glandular enlargement and the symptoms of an undoubted general infection with manifestations of the skin and mucous membranes. I might state here that mistakes are sometimes made in the diagnosis of a second initial lesion, by confounding an indurated papule with it. A papule making its appearance on the corona is very apt to become indurated on account of the continued irritation; on this situation any simple little sore or abrasion may become indurated, because of the greatly increased vascular supply. As an example I will cite a case:

A man, T., came to me in 1895, having acquired ordinary chancroids; in a few days one of them, situated directly upon the corona, became markedly indurated. At that time I expressed the opinion that he probably had a mixed infection, but, contrary to my expectation, the sore healed without an untoward symptom. In August of the following year he presented himself again with a typical primary sore, which was followed in due time by a macular eruption and sore throat.

Many cases of reinfection have been reported by different observers, which leave no doubt as to the possibility of the cure of syphilis. I have seen two cases of undoubted second infection, which occurred at intervals of six and three years respectively. Diday recorded twenty cases of reinfection in six years and mentions one case in which four reinfections occurred. Cooper had seen five cases of reinfection in one year. If the vast amount of medical literature were searched, many cases of reinfection would be found; as it is, the most reliable sources of information are in hospitals, where complete records of all cases are kept. It has been stated that when the interval between infections was short, the disease pursued a mild course and that when a long period elapsed between infections, the symptoms were more severe. This may be to a certain extent true, for syphilis occurring in a young healthy person is more apt to pursue a milder course than it does in a person of more advanced years, where the vitality is lower and the constitution undermined by previous excesses; for syphilis occurring in such persons is apt to be very intractable and often assumes the malignant type Again, it has been held by many that though syphilis can be cured, yet when it reaches the tertiary stage, it then becomes incurable. Yet Gascoyne in his number of cases of reinfection, reports that six were suffering from tertiary symptoms at the time of reinfection. This, however, brings up another question in connection with syphilis, which is that tertiary symptoms are sequelæ, rather than symptoms of syphilis. This, however, has no relation with the subject at hand.

As the time allotted to the reading of papers is, I understand, to be limited, I have made this paper very short, for much might be said on the subject and many cases cited and writers quoted. One word in conclusion: Always begin the treatment as early as possible; that is, as soon as the diagnosis is certain and impress upon the patient that the disease can be cured only by a prolonged course of treatment and good hygiene.

362 PEARL STREET.

